# Ketogenic Diet Improves Motor Function and Motor Unit Connectivity in Aged C57BL/6 Mice

**DOI:** 10.21203/rs.3.rs-3335211/v1

**Published:** 2023-10-27

**Authors:** Carlos J Padilla, Hallie Harris, Jeff S Volek, Brian C Clark, W. David Arnold

**Affiliations:** University of Wisconsin-Madison; The Ohio State University; The Ohio State University; Ohio University; University of Missouri

**Keywords:** Ketogenic diet, Motor function, Motor units number estimation, Aging

## Abstract

**Objective:**

Pathological, age-related loss of muscle function, commonly referred to as sarcopenia, contributes to loss of mobility, impaired independence, as well as increased risk of adverse health events. Sarcopenia has been attributed to changes in both neural and muscular integrity during aging. Current treatment options are primarily limited to exercise and dietary protein fortification, but the therapeutic impact of these approaches are often inadequate. Prior work has suggested that a ketogenic diet (KD) might improve healthspan and lifespan in aging mice. Thus, we sought to investigate the effects of a KD on neuromuscular indices of sarcopenia in aged C57BL/6 mice.

**Design:**

A randomized, controlled pre-clinical experiment consisting of longitudinal assessments performed starting at 22-months of age (baseline) as well as 2, 6 and 10 weeks after the start of a KD vs. regular chow intervention.

**Setting:**

Preclinical laboratory study.

**Sample size:**

Thirty-six 22-month-old mice were randomized into 2 dietary groups: KD [n = 22 (13 female and 9 male)], and regular chow [n = 15 (7 female and 8 male)].

**Measurements:**

Measures included body mass, hindlimb and all limb grip strength, rotarod for motor performance, plantarflexion muscle contractility, motor unit number estimations (MUNE), and repetitive nerve stimulation (RNS) as an index of neuromuscular junction transmission efficacy recorded from the gastrocnemius muscle. At end point, blood samples were collected to assess blood beta-hydroxybutyrate levels.

**Statistical Analysis:**

Two-way ANOVA mixed-effects analysis (time x diet) were performed to analyze grip, rotarod, MUNE, and muscle contractility data.

**Results:**

Beta-hydroxybutyrate (BHB) was significantly higher at 10 weeks in mice on a KD vs control group (0.83 ± 0.44 mmol/l versus 0.42 ± 0.21 mmol/l, η^2^ = 0.265, unpaired t-test, p = 0.0060). Mice on the KD intervention demonstrated significantly increased hindlimb grip strength (time x diet, p = 0.0030), all limb grip strength (time x diet, p = 0.0523), and rotarod latency to fall (time x diet, p = 0.0021). Mice treated with the KD intervention also demonstrated significantly greater MUNE (time x diet, p = 0.0064), but no difference in muscle contractility (time x diet, p = 0.5836) or RNS (time x diet, p = 0.9871).

**Conclusion:**

KD intervention improved neuromuscular and motor function in aged mice. This pre-clinical work suggests that further research is needed to assess the efficacy and physiological effects of a KD on indices of sarcopenia.

## INTRODUCTION

Muscle function in older adults is critical for maintaining independence in daily life, and impaired physical function is associated with adverse health outcomes and increased risk of mortality ^1^. Age-related decline of muscle function has been attributed to changes in both the nervous and muscular systems ^2^. Prior work has indicated that there is progressive decline in muscle function (e.g., strength) beginning around 30 years of age with an accelerated loss after 60 years of age ^3,4^. The age-related reduction in muscle strength is notably greater than the loss of muscle mass ^5^, which indicates that other changes in the neuromuscular system beyond atrophy are mechanistically associated with the loss of muscle function ^6,7^. As such, it is not surprising that poor motor nerve function is linked to muscle weakness and mobility disability ^8–11^.

Resistance exercise is the mainstay treatment and prevention strategy for sarcopenia ^12^. However, most older adults do not perform resistance exercise training ^13^, and even among those that do there is a large degree of between person variability in the strength response observed ^14,15^. Optimized nutrition has been suggested by many to offer potential benefits for aging ^16–21^, and studies have suggested that a ketogenic diet (KD) may improve muscle performance and maintenance in the context of both sarcopenia ^22^ and cachexia ^23^. The KD is characterized by high fat intake (60–90%), modest protein intake (10–20%), and very low carbohydrate intake (5–10%, usually < 50 g/day) of the total energy content of the diet. From a physiological perspective, the KD results in an increase in plasma ketone bodies, especially β-hydroxybutyrate (BHB), the most abundant ketone found in the circulation ^24^. Neuroketotherapy represents a class of bioenergetic medicine therapies that are characterized by the induction of ketosis ^25^. The KD has shown protective effects in a broad spectrum of neurological disorders, including models of neurodegeneration, neurogenetic conditions, cerebrovascular disease, and immune-mediated demyelination ^26–28^.

Accordingly, in the current study, we sought to investigate the effects of a KD on neuromuscular indices of sarcopenia in aged C57BL/6 mice. Specifically, in building on findings that suggest that the KD has positive effects on both muscles and nerves ^29,30^, we were interested in determining the effects and consequences of a KD on motor unit, neuromuscular junction, and muscle contractile function.

## METHODS

### Experimental Overview

**Figure 1:**
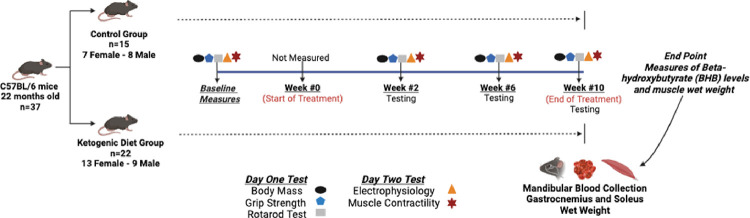
Study design. Thirty-six 22-month-old mice were randomized into 2 groups: a group fed a ketogenic diet ad libitum for 10 weeks (Envigo Teklad TD.96355; 9.2% protein, 0.3% carbohydrates and 90.5% fat) [n=22 (13 female and 9 male)] and a group fed regular chow ad libitum for 10 weeks (Envigo Teklad 7012; 25% protein, 58% carbohydrates and 17% fat) [n=15 (7 female and 8 male)]. Body mass, electrophysiology, muscle contractility, grip strength, and motor performance were measured at baseline as well as after 2, 6, and 10 weeks of the intervention. At endpoint (week 10), ketone blood levels (Beta – Hydroxybutyrate, mmol/l) were assessed using blood obtained by a mandibular bleed and gastrocnemius and soleus muscle wet weights.

All procedures were approved and performed in accordance with the Institutional Animal Care and Use Committee at The Ohio State University. Aged (22 months) C57BL/6 mice were assessed at baseline and then randomized to a KD (n = 22, 13 female and 9 male) or regular chow diet (n = 15, 7 female and 8 male) ad libitum. Longitudinal assessments were performed at baseline as well as 2, 6 and 10 weeks after the start of the respective dietary intervention. Outcome measures included body mass, hindlimb and all limb grip strength, rotarod latency to fall to assess motor performance, electrically stimulated plantarflexion muscle contractility. Additionally, in the gastrocnemius muscles we used the incremental stimulation motor unit number estimate (MUNE) technique to assess the number of functioning motor units ^31^, as well as repetitive nerve stimulation at 50 Hz to assess neuromuscular junction transmission ^32^. At end point (week 10 post intervention), blood samples were collected (submandibular bleed) to assess blood ketone levels (Beta-hydroxybutyrate-BHB). The study design is presented in Fig. 1.

### Diet Intervention

The dietary interventions were administered in the morning as needed during the 10-week study. Diets were prepared by Envigo Teklad Diets (Madison, WI) and placed in a small container inside the mouse cages. The High-Fat Ketogenic Teklad Custom Diet (TD.96355) used a fat to protein + carbohydrate ratio. For the regular chow, the 7012 Teklad LM-485 Mouse Sterilizable Diet was used, which is a fixed formula, autoclavable diet, manufactured with high quality ingredients and designed to support the growth of rodents. Typical concentrations of isoflavones (daidzein + genistein aglycone equivalents) range from 300 to 600 mg/kg. 7012 is supplemented with additional vitamins to ensure nutritional adequacy after autoclaving. The ingredients used were: Ground corn, dehulled soybean meal, ground oats, wheat middling, dehydrated alfalfa meal, soybean oil, corn gluten meal, calcium carbonate, dicalcium phosphate, brewers dried yeast, iodized salt, choline chloride, kaolin, magnesium oxide, L-lysine, DL-methionine, ferrous sulfate, menadione sodium bisulfite complex (source of vitamin K activity), vitamin E acetate, thiamin mononitrate, calcium pantothenate, manganous oxide, niacin, copper sulfate, zinc oxide, vitamin A acetate, pyridoxine hydrochloride, riboflavin, vitamin D3 supplement, vitamin B12 supplement, folic acid, biotin, calcium iodate, and cobalt carbonate. Nutrient information and values for the ketogenic diet and regular chow were selected and calculated from ingredient analysis and manufacturer data. The dietary information is presented in the Table 1.

### Animal Anesthesia and Preparation

Mice were anesthetized during electrophysiological recordings and during the muscle contractility assessment via inhaled isoflurane delivered at 3–5% for induction and 1–2% for maintenance anesthesia using a SomnoSuite low-flow anesthesia system (Kent Scientific, Torrington, CT). Body temperature was maintained at 37°C with an infrared heating pad (Kent Scientific). To avoid corneal dryness, a petroleum-based eye lubricant (Dechra, Northwich, UK) was applied. Hair of the right hindlimb was shaved (model VPG 6530, Remington, DeForest, WI). The procedures were carried out as we have previously described ^31,33,34^.

### Grip Strength Test

Bilateral hindlimb and all limb grip strength were assessed as previously described using a force transducer (Model GT3, Bioseb SAS BP32025-F-13845 Vitrolles Cedex, Pinellas Park, FL, USA) ^35^. Mice were grasped and allowed to grip a T-shaped bar connected to the transducer and then were pulled away from the grip meter using a steady and constant motion until grip was lost. For all limb grip, mice were allowed to grip a grid connected to the force transducer and were pulled away from the grip meter using a steady and constant motion until grip was lost. Three trials of hindlimb and all limb grip strength were completed, and the average of the three trials (in grams) was used for analyses.

### Rotarod Latency to Fall

Coordination and motor performance were analyzed and conducted using the rotarod test (BX-ROD, Bioseb). To start the test, once the mice were placed on the rod, the rod started rotating at 4 rpm and accelerated at 1 rpm/6 s to a maximum of 40 rpm ^36^. Three trials were performed at each timepoint and averaged.

### Electrophysiological Assessment of Motor Unit Size and Number and Neuromuscular Junction Transmission

Motor Unit Number Estimation (MUNE) was performed to estimate the number of functioning motor units using an approach similar to previous studies in aged mice via a clinical electrodiagnostic system (Cadwell, Kennewick, WA, USA) ^31,33^. A pair of 28-gauge insulated monopolar needle electrodes (Teca, Oxford Instruments Medical, NY, USA) were inserted subcutaneously into the proximal hindlimb in the region of the sciatic nerve as the cathode and anode for stimulation. A pair of fine wire ring electrodes (Alpine Biomed, Skovlunde, Denmark) were used as the active electrode (placed over proximal gastrocnemius just distal to the knee) and reference electrode (placed over the metatarsal area of the right foot). The ground electrode was placed on the tail (Care Fusion, Middleton, WI, USA). Low frequency and high frequency filters were set at 10 Hz to 10 kHz, respectively. To determine MUNE, first, the peak-to-peak amplitude of the compound muscle action potential was recorded following supramaximal stimulation of the sciatic nerve (constant current: <10mA, duration 0.1 ms). Then, 10 incremental, all-or- none responses obtained during a gradually increasing stimulations were recorded and averaged to calculate the average single motor unit potential (SMUP) amplitude. Then, MUNE was calculated as such: MUNE = Peak-to-peak CMAP Amplitude/ SMUP.

Repetitive Nerve Stimulation (RNS) testing was then performed using the same recording setup as described for MUNE. During RNS, trains of 10 stimulations were delivered at 50 Hz. Amplitude changes between the first and 10th stimulations were calculated using the following formula: % amplitude decrease= [(Amplitude of 10th response − Amplitude 1st response)/Amplitude of 1st response] * 100%.

### Plantar Flexion Muscle Contractility

For muscle contractility testing, mice were placed in supine on the testing platform to assess the right hindlimb using an in vivo muscle contractility system (Aurora Scientific Inc, Canada Model 1300A Muscle). The right hindlimb was taped to a rotating foot plate connected to a dual control motor to assess plantar flexion torque. Then, the hindlimb was locked into testing frame connected to the platform base using blunt clamps at the femoral condyles taking care to avoid injury of the fibular nerve at the fibular head. The tibial nerve located in the posterior medial knee was then stimulated using two insulated monopolar electrodes placed subcutaneously (Natus Neurology Inc, Middleton, WI, USA). Stimulation was adjusted (constant current: 0–10 mA, 0.2 ms) to determine the intensity required for a maximal twitch response and adjusted to 120–150% to ensure maximal stimulation. Peak twitch torque was measured after a single 0.2 ms supramaximal pulse-wave stimulation. Tetanic torque was measured using a 200 ms train of stimuli delivered at 125 Hz. This entire process was carried out as previously described ^31,33,37^.

### Blood Collection

Blood beta-hydroxybutyrate and blood glucose were assessed using the Keto mojo B Ketone and Blood Glucose Monitoring System Test Kit. Blood was obtained from the submandibular vein at week 10. The animal did not need to be anesthetized for this process. The mouse was held in one hand using the index finger and thumb which applied the desired pressure to the maxillary vein. The maxillary vein was located along the curvature of the mandible, just below this mark in the groove that runs through the mandible. Using a lancet (needle), firm pressure was applied to the maxillary point, caudal to the eye and ventral to the ear, where the submandibular vein is located, then released until blood flowed. The lancet was kept perpendicular to the bleeding site to avoid injuring the ear canal. Ketone and glucose test strips were then placed under the puncture site using the Keto – Mojo GK + blood glucose & B – ketone dual monitoring system instrument until the desired volume of blood was collected for measurement in mmol/L. Finally, gentle pressure was applied with a gauze over the mandibular area to stop the bleeding of the mouse.

### Wet Weight

At the end of the study, the mice were deeply anesthetized before being sacrificed. The gastrocnemius and soleus muscles of the right hindlimb were dissected and removed. Wet weight in grams was recorded on a previously calibrated scale.

### Statistical Analyses

Statistical analyzes were performed using the GraphPad Prism 9.5.0 program (GraphPad Software Inc., San Diego, CA, USA). To identify whether there was a trend for an effect of the intervention on body mass, the Eta^2^ effect size ( ^2^) was used. For the grip test, rotarod performance test, electrophysiology and muscle contractility analyses, two-way ANOVA mixed-effects analysis (time x diet) was used to compare the dietary groups across time points. To determine group differences in blood ketone levels in the intervention group and control group, an unpaired t-test was used. Statistical significance was set at p < 0.05.

## RESULTS

### KD significantly increases blood ketones but shows minimal impact on body mass

**Figure 2:**
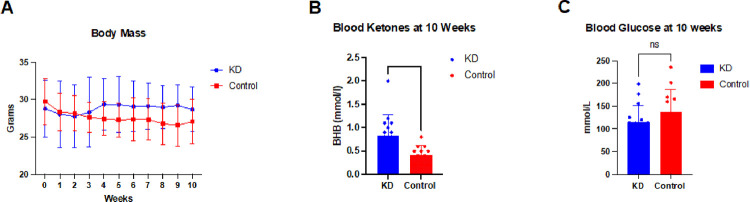
Body mass, beta-hydroxybutyrate and glucose levels. **(A)** Body mass, beta-hydroxybutyrate and glucose levels. (A) Body mass showed a moderate effect for increasing in the ketogenic diet (KD) group relative to the control group (h^2^ =0.422), but this effect did not reach statistical significance (Mixed-effects analysis two-way ANOVA time x diet, p=0.0882). hydroxybutyrate (BHB) was significantly higher at 10 weeks in mice on a KD vs control group Beta-(0.8333±0.44 mmol/l versus 0.42±0.21 mmol/l, h^2^ =0.265, unpaired t-test, p=0.0060). Blood glucose at 10 weeks showed a moderate reduction in the ketogenic diet group versus control group, but this effect did not reach statistical significance (115.1±37.11 mmol/L/ versus 138±49.11 mmol/L, h^2^ =0.070, unpaired t-test, p=0.1800). **p<0.01

Body mass in C57BL/6 mice treated with a KD did not demonstrate a statistically significant increase in comparison to the control group although a moderate effect size was observed (η^2^ = 0.422) (Fig. 2A). As expected, mice treated with a KD showed a 2-fold greater level of BHB at 10 weeks (p = 0.0060) (Fig. 2B). In addition, the group of mice treated with a KD showed a slight but not significant reduction in blood glucose at 10 weeks of study (η^2^ = 0.070).

### Motor function and motor unit integrity are improved in aged mice fed a ketogenic diet

**Figure 3:**
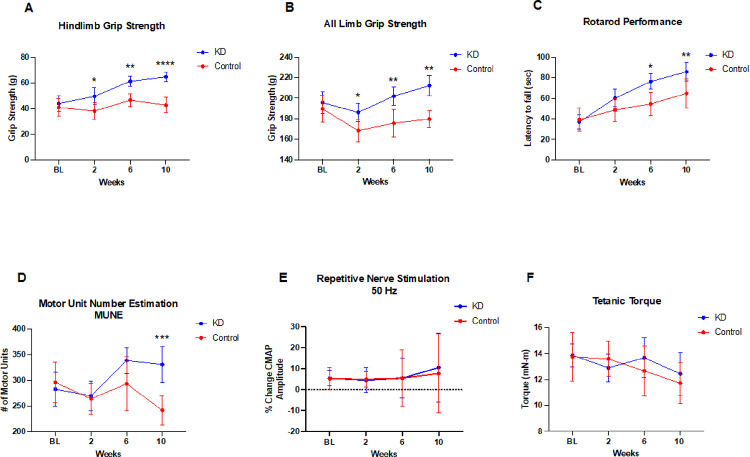
Aged mice fed a ketogenic diet show improved motor function and motor unit number. Mice in the KD group demonstrated significance increases in **(A)** Hindlimb grip strength (Mixed-effects analysis two-way ANOVA time x diet, p=0.0030); **(B)** All limb grip (Mixed-effects analysis two-way ANOV time x diet, p=0.0523) and **(C)**Rotarod latency to fall (Mixed-effects analysis two-way ANOVA time x diet, p=0.0021). Mice in the KD group also demonstrated significance increase in **(D)** Motor unit number estimation (MUNE) (Mixed-effects analysis two-way ANOVA time × diet, p=0.0064) but not in **(E)** peak tetanic muscle contractility torque at 150 Hz stimulation at baseline to 10 weeks (Mixed-effects analysis two-way ANOVA time × diet, p=0.5836) or **(F)** repetitive nerve stimulation at 50 Hz recorded at baseline to 10 weeks (Mixed-effects analysis two-way ANOVA time × diet, p=0.9871). Plot values are mean with 95% CI. Šídák’s multiple comparisons test adjust p values: *p<0.05, **p<0.01, ***p<0.001, ****p<0.0001. BL= baseline.

Both hindlimb and all limb grip strength were significantly improved in mice treated with a KD compared with a control group (time x diet p = 0.0030–0.0523) (Fig. 3A–B). Specifically, at endpoint the hindlimb grip showed a 36% increase in the KD group versus 3% in the control group, and all-limb grip showed a 6% increase in the KD group versus 5% decrease in the control group. Similarly, rotarod, a measure of motor stamina and coordination, was improved in mice on the KD showing of 78% increase over the 10-weeks study versus 48% increase in the control group (time x diet, p = 0.0021) (Fig. 3C).

MUNE is an electrophysiological technique that can be used to monitor the functional status of a motor unit pool in vivo ^11^. MUNE was higher in mice of the KD group showing a 16% increase at 10 weeks of study versus a −20% reduction in the control group (time × diet, p = 0.0064) (Fig. 3D). RNS, a measure of neuromuscular junction transmission, did not exhibit a significant time × diet interaction (p = 0.9871) (Fig. 3E).

### No differences for muscle contractility with KD intervention

In contrast to measures of motor and motor unit function, the changes in muscle contractility was similar between aged mice in the KD and regular chow groups (p = 0.5836) (Fig. 3E).

### Ketogenic diet did not show effects on the wet weight of gastrocnemius and soleus

**Figure 4:**
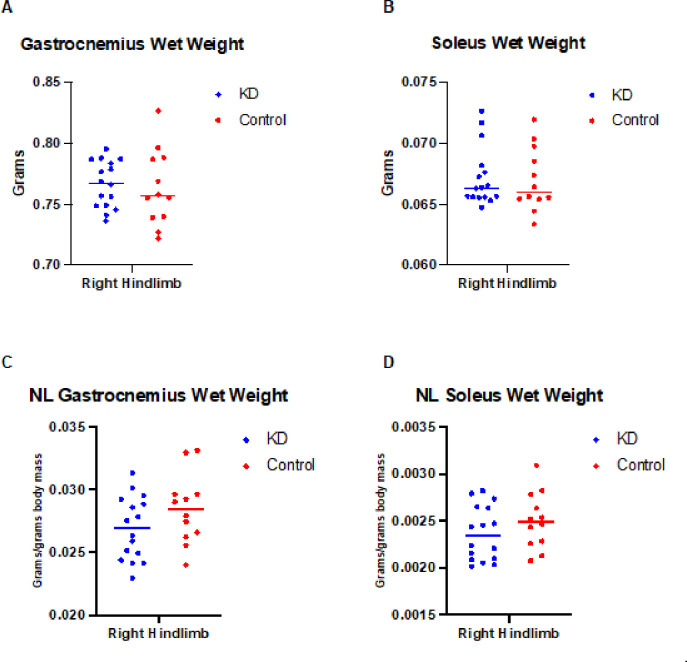
Ketogenic diet on gastrocnemius and soleus wet weight and normalized (NL) in aged C57BL/6 mice. No significant changes in gastrocnemius and soleus wet weight (whole) and normalized by body mass were observed after consumption of the ketogenic diet ad libitum for 10 weeks. **(A)** Mean ± SD for gastrocnemius wet weight in the KD group was 0.7621 ± 0.0190 and for the control group 0.7636 ± 0.0310 (h^2^ =0.003, unpaired t-test, p=0.7621). **(B)** For the soleus wet weight in the KD group it was 0.0672 ± 0.0024 and for the control group 0.0670 ± 0.0026 (h^2^ =0.001, unpaired t-test, p=0.8497). **(c)** Mean ± SD for NL gastrocnemius wet weight in the KD group was 0.0269 ± 0.0025 and for the control group 0.0284 ± 0.0027 (h^2^ =0.080, unpaired t-test, p=0.1434). **(B)** NL soleus wet weight in the KD group it was 0.0023 ± 0.0002 and for the control group 0.0025 ± 0.0002 (h^2^ =0.052, unpaired t-test, p=0.2402).

The ketogenic diet intervention was evaluated for 10 weeks in C57BL/6 mice at 25 months of age on the wet weight of the gastrocnemius (p = 0.7621, η^2^ = 0.003), and soleus (p = 0.8497, η^2^ = 0.001) muscles on the right side of the hindlimb, with no group differences observed in the results (Fig. 4A and 4B). Similarly, no significant differences were observed in muscles normalized by body mass, gastrocnemius, p = 0.1434, η^2^ = 0.080 and soleus, p = 0.2402, η^2^ = 0.052 (Fig. 4C and 4D).

## DISCUSSION

In the present study, we examined the effect of 10 weeks of a KD on motor function and indices of sarcopenia in aged C57BL/6 mice. There is building evidence that integrity of the nervous system is a major contributor to sarcopenia in older adults ^38^. Despite this, few interventions, including nutritional, have been investigated for improving sarcopenia through neurological mechanisms. It was previously reported that the KD increases lifespan in male mice by 13.6% and preserved physical function ^22^. Our study adds to this body of knowledge as it demonstrates that a KD intervention improves motor function and enhances motor unit function in aged mice.

### KD improved muscle strength and motor performance during aging in mice

Our results show that the 10-week KD intervention significantly and progressively improved hindlimb and all-limb grip strength. Similar results were found by Roberts et al. (2017) where, after a KD intervention, aged mice showed greater grip strength compared to those in the control group ^22^. Other studies by Ahola-Erkkila et al. (2010) also showed an effect of the KD in the context of other forms of muscle weakness including a mouse model of late-onset mitochondrial myopathy ^39^. It is important to note that this prior work indicated that the KD intervention enhances muscle strength even in the absence of a physical/exercise stimulus. For example, in a study by Camajani et al. (2022) using adults that were 50 to 70 years of age who undertook a KD without physical/exercise training showed a significant increase in muscle strength and function ^40^. These results demonstrate the potential of the KD intervention to improve muscle strength during aging. Another study carried out by Zhou et al. (2023) also demonstrated the ability of the KD on forelimb muscle strength under isometric contraction in the grid-wire suspension test during aging in male mice ^41^. It has been suggested that the reason a KD enhances muscle function in the context of aging is due to the availability of ketone bodies (BHB), which provide energy substrates ^22,29^.

In our study, mice fed with the KD showed significant increase in the motor performance on grip strength testing and latency to fall during rotarod testing. Beckett et al. (2013) previously demonstrated that APP/PS1 knock-in mice fed a ketogenic diet ad libitum for one month (endpoint 2–3 months old) showed better rotarod performance ^42^. Other work has shown that the KD can improve motor performance, and that exogenous ketone supplementation in rodents may be a viable alternative for those who cannot consume a KD ^43^. For instance, a study using a 5-month-old mouse model of Alzheimer’s disease fed for 16 weeks on the KD showed that the average latency to fall in the accelerating rotarod was significantly higher in the KD-fed mice compared to the control group ^44^. In summary, these results, as well as those of our study, suggest that the ketogenic diet could play an important role in improving motor performance in aging mice.

### Motor unit number was higher in aged mice fed a ketogenic diet

The number of functional motor units that innervate a muscle is a fundamental element for neuromuscular control, and losses of motor units is considered a potentially important factor in the development of sarcopenia ^31,45,46^. Prior work has shown that estimates of motor unit numbers using MUNE are reduced in older adults ^47–49^. Here we report novel findings where the KD intervention improved motor unit number in aged mice compared with mice on a regular chow. Similar to our findings in wild-type mice, a study by Zhao et al. (2006) reported that KD administration resulted in increased motor neuron survival and improved motor function in a G93A-SOD1 transgenic mouse model. This could be relevant to our study as it could explain how the ketogenic diet helps to improve and increase the motor units’ number that are lost during aging. The results from Zhao et al. (2006) are supported by the fact that the ketogenic diet-induced elevation of blood ketones could ameliorate mitochondrial defects by increasing mitochondrial function and ATP production ^50^. The results of Zhao and our study suggest that the KD intervention may improve muscle fiber innervation of an alpha motor neuron, triggering better function of the neuromuscular system during aging. Based on our study results the physiological effect of the ketogenic diet on MUNE may have been the result of increased plasma ketone bodies, especially BHB, the most abundant ketone found in circulation. Based on prior work, increase of ketones bodies may improve mitochondrial respiration, promote long-term neuronal potentiation, increase expression of brain-derived neurotrophic factor, increase G-coupled protein receptor signaling, attenuate oxidative stress, reduce inflammation, and alter post-translational modifications of the protein through lysine acetylation and BHB ^25^.

More studies are needed to clearly establish the mechanisms of the KD on the number of motor units in advanced ages. KD can cause some molecular changes to potentiate neuronal plasticity ^51^. One possibility is that KD induced plasticity may promote improved innervation and thus increase of MUNE during aging. For example, KD increases serum leptin levels as early as 5 days after starting the diet ^52^ and leptin is known to be not only neuroprotective but also to enhance neuronal plasticity in vitro and in vivo ^53,54^. Additionally, the stimulatory effect of BHB on mitochondrial density could facilitate neuronal plasticity. Dendritic mitochondria have been implicated in synapse formation and the number of mitochondria correlates with the number of newly formed synapses ^55–57^. So, this result could have been a factor to cause that in our study the motor unit number that innervated the gastrocnemius muscle increased during aging.

### Muscle contractility was not improved by the ketogenic diet

The intrinsic force producing capacity of skeletal muscle decreases with advancing age in humans and mice ^58^. The soleus muscle of female C57BL/6 mice demonstrates a reduction in maximal tetanic force ~ 4, 8-, 16-, 24-, and 28-months during aging but muscle size and contractile protein content were not affected ^59^. This may have been the result of the authors being unable to clearly explain the reduction in strength that occurs with aging. Furthermore, it should be noted that preservation of motor function through KD has been associated with higher relative weights in hindlimb muscles in aged mice. However, our study did not show significant increases with the intervention of the ketogenic diet in gastrocnemius and soleus wet weight during aging. In our study we wanted to examine the effect of the KD (~ 90% fat and 10% protein) on muscle contractility during aging in C57BL/6 mice. The results show that tetanic torque was not affected by the KD intervention for 10 weeks. Similarly, previous studies showed that feeding the KD for 4 weeks did not affect tetanic contraction in aged male rats ^60^. According to the results of our study, the KD increased hindlimb and all limb muscle strength but did not affect gastrocnemius muscle contractility during aging. These results support the notion that the KD intervention improves maximal limb strength through neurological mechanisms since there is no effect on the intrinsic tetanic torque capacity of the plantar flexors muscles.

## CONCLUSIONS

In this study we establish that the KD improves motor function in aged mice and demonstrate that this improved function may be related to improved motor unit connectivity with muscle. We observed that the 10-weeks ketogenic diet intervention improved muscle strength, motor performance, and increased the number of functioning motor units, but had no effect on muscle contractility, mass, and/or an index of neuromuscular transmission during aging. These results strengthen previous research on the potential of KD therapies to improve motor function during aging and support the potential of a KD to improving or maintaining function in older adults.

## LIMITATIONS

There are several limitations of this work that should be noted. First, we used the ketogenic diet to increase ketone bodies in the study mice (9.2% protein, 0.3% carbohydrates and 90.5% fat) but did not quantify the amount of food or calorie intake ingested by the mice (5 mice per cage). Second, we did not track ketosis status during the study to examine the relationship between the degree of ketosis and functional outcomes. Third, while we used male and female mice the study was not powered to permit examination of sex-specific effects. Thus, further work is needed that more fully considers sex as a biological variable. Lastly, future studies examining the differential effects of ketogenic (endogenous) and ketone esters (exogenous) diets are also needed to clearly identify the physiological effects and consequences of each intervention.

## Figures and Tables

**Table 1:** Macronutrient composition, energy density and calories from macronutrients.

Macronutrients		Regular Chow (7012 Teklad LM-485)	KD (TD.96355)
Protein	%	19.1	15.3
Carbohydrate	%	44.3	0.5
Fat	%	5.8	67.4
Fiber	%	4.6	8.8
**Energy Density**	kcal/g	3.1	6.7
**Calories From:**			
Protein	%	25	9.2
Carbohydrate	%	58	0.3
Fat	%	17	90.5

## References

[1] Kutsuna T, Hiyama Y, Kusaka S, The effect of short-term health promotion intervention on motor function in community-dwelling older adults. Aging Clin Exp Res. 2019;31(4). doi:10.1007/s40520-018-0994-x 29971630

[2] Wu R, Ditroilo M, Delahunt E, De Vito G. Age Related Changes in Motor Function (II)Decline in Motor Performance Outcomes. Int J Sports Med. 2021;42(3). doi:10.1055/a-1265-7073 33137831

[3] Bongard V, McDermott AY, Dallal GE, Schaefer EJ. Effects of age and gender on physical performance. Age (Omaha). 2007;29(2–3). doi:10.1007/s11357-007-9034-z PMC2267663 19424833

[4] Santos PCR dos, Lamoth CJC, Gobbi LTB, Zijdewind I, Barbieri FA, Hortobágyi T. Older Compared with Younger Adults Performed 467 Fewer Sit-to-Stand Trials, Accompanied by Small Changes in Muscle Activation and Voluntary Force. Front Aging Neurosci. 2021;13. doi:10.3389/fnagi.2021.679282 PMC8276699 34267644

[5] Goodpaster BH, Park SW, Harris TB, The loss of skeletal muscle strength, mass, and quality in older adults: The Health, Aging and Body Composition Study. Journals of Gerontology - Series A Biological Sciences and Medical Sciences. 2006;61(10). doi:10.1093/gerona/61.10.1059 17077199

[6] Hunter SK, Pereira XHM, Keenan KG. The aging neuromuscular system and motor performance. J Appl Physiol. 2016;121(4). doi:10.1152/japplphysiol.00475.2016 PMC5142309 27516536

[7] Clark BC, Carson RG. Sarcopenia and Neuroscience: Learning to Communicate. Journals of Gerontology - Series A Biological Sciences and Medical Sciences. 2021;76(10). doi:10.1093/gerona/glab098 PMC8436984 33824986

[8] Piasecki M, Ireland A, Piasecki J, Stashuk DW, McPhee JS, Jones DA. The reliability of methods to estimate the number and size of human motor units and their use with large limb muscles. Eur J Appl Physiol. 2018;118(4). doi:10.1007/s00421-018-3811-5 PMC5843678 29356950

[9] Deschenes M R.. Motor Unit and Neuromuscular Junction Remodeling with Aging. Current Aging Sciencee. 2012;4(3). doi:10.2174/1874609811104030209 21529328

[10] Piasecki M, Ireland A, Coulson J, Motor unit number estimates and neuromuscular transmission in the tibialis anterior of master athletes: evidence that athletic older people are not spared from age-related motor unit remodeling. Physiol Rep. 2016;4(19). doi:10.14814/phy2.12987 PMC5064139 27694526

[11] Arnold WD, Sheth KA, Wier CG, Kissel JT, Burghes AH, Kolb SJ. Electrophysiological motor unit number estimation (MUNE) measuring compound muscle action potential (CMAP) in mouse hindlimb muscles. Journal of Visualized Experiments. 2015;2015(103). doi:10.3791/52899 PMC4676269 26436455

[12] McPhee JS, French DP, Jackson D, Nazroo J, Pendleton N, Degens H. Physical activity in older age: perspectives for healthy ageing and frailty. Biogerontology. 2016;17(3). doi:10.1007/s10522-016-9641-0 PMC4889622 26936444

[13] Tavoian D, Russ DW, Consitt LA, Clark BC. Perspective: Pragmatic Exercise Recommendations for Older Adults: The Case for Emphasizing Resistance Training. Front Physiol. 2020;11. doi:10.3389/fphys.2020.00799 32719618 PMC7348658

[14] Karavirta L, Häkkinen K, Kauhanen A, . Individual responses to combined endurance and strength training in older adults. Med Sci Sports Exerc. 2011;43(3). doi:10.1249/MSS.0b013e3181f1bf0d 20689460

[15] Clark LA, Russ DW, Tavoian D, Heterogeneity of the strength response to progressive resistance exercise training in older adults: Contributions of muscle contractility. Exp Gerontol. 2021;152. doi:10.1016/j.exger.2021.111437 PMC8319076 34098008

[16] Schneider DA, Trence DL. Possible role of nutrition in prevention of sarcopenia and falls. Endocrine Practice. 2019;25(11). doi:10.4158/EP-2019-0144 31412231

[17] Kobayashi H. Amino acid nutrition in the prevention and treatment of sarcopenia. Yakugaku Zasshi. 2018;138(10). doi:10.1248/yakushi.18-00091-4 30270272

[18] Woo J, Ong S, Chan R, Nutrition, sarcopenia and frailty: An Asian perspective. Transl Med Aging. 2019;3. doi:10.1016/j.tma.2019.11.001

[19] Robinson SM, Reginster JY, Rizzoli R, Does nutrition play a role in the prevention and management of sarcopenia? Clinical Nutrition. 2018;37(4). doi:10.1016/j.clnu.2017.08.016 PMC5796643 28927897

[20] Wakabayashi H. Role of nutrition and rehabilitation in the prevention and management of sarcopenia and frailty. In: Recent Advances of Sarcopenia and Frailty in CKD. ; 2020. doi:10.1007/978-981-15-2365-6_8

[21] Bhattacharya S, Bhadra R, Schols AMWJ, van Helvoort A, Sambashivaiah S. Nutrition in the prevention and management of sarcopenia - A special focus on Asian Indians. Osteoporos Sarcopenia. 2022;8(4). doi:10.1016/j.afos.2022.12.002 PMC9805983 36605171

[22] Roberts MN, Wallace MA, Tomilov AA, A Ketogenic Diet Extends Longevity and Healthspan in Adult Mice. Cell Metab. 2017;26(3). doi:10.1016/j.cmet.2017.08.005 PMC5609489 28877457

[23] Koutnik AP, Poff AM, Ward NP, Ketone Bodies Attenuate Wasting in Models of Atrophy. J Cachexia Sarcopenia Muscle. 2020;11(4). doi:10.1002/jcsm.12554 PMC7432582 32239651

[24] Valenzuela PL, Castillo-garcía A, Lucia A, Naclerio F. Effects of combining a ketogenic diet with resistance training on body composition, strength, and mechanical power in trained individuals: A narrative review. Nutrients. 2021;13(9). doi:10.3390/nu13093083 PMC8469041 34578961

[25] Koppel SJ, Swerdlow RH. Neuroketotherapeutics: A modern review of a century-old therapy. Neurochem Int. 2018;117. doi:10.1016/j.neuint.2017.05.019 PMC5711637 28579059

[26] Benlloch M, López-Rodríguez MM, Cuerda-Ballester M, Satiating effect of a ketogenic diet and its impact on muscle improvement and oxidation state in multiple sclerosis patients. Nutrients. 2019;11(5). doi:10.3390/nu11051156 PMC6566517 31126118

[27] Xu K, Ye L, Sharma K, Diet-induced ketosis protects against focal cerebral ischemia in mouse. Adv Exp Med Biol. 2017;977. doi:10.1007/978-3-319-55231-6_28 28685447

[28] Puchalska P, Crawford PA. Multi-dimensional Roles of Ketone Bodies in Fuel Metabolism, Signaling, and Therapeutics. Cell Metab. 2017;25(2). doi:10.1016/j.cmet.2016.12.022 PMC5313038 28178565

[29] Wallace MA, Aguirre NW, Marcotte GR, The ketogenic diet preserves skeletal muscle with aging in mice. Aging Cell. 2021;20(4). doi:10.1111/acel.13322 PMC8045940 33675103

[30] Paoli A, Bianco A, Damiani E, Bosco G. Ketogenic diet in neuromuscular and neurodegenerative diseases. Biomed Res Int. 2014;2014. doi:10.1155/2014/474296 PMC4101992 25101284

[31] Sheth KA, Iyer CC, Wier CG, Muscle strength and size are associated with motor unit connectivity in aged mice. Neurobiol Aging. 2018;67. doi:10.1016/j.neurobiolaging.2018.03.016 29656012 PMC5981861

[32] Juel VC. Evaluation of neuromuscular junction disorders in the electromyography laboratory. Neurol Clin. 2012;30(2). doi:10.1016/j.ncl.2011.12.012 22361377

[33] Chugh D, Iyer CC, Wang X, Bobbili P, Rich MM, Arnold WD. Neuromuscular junction transmission failure is a late phenotype in aging mice. Neurobiol Aging. 2020;86. doi:10.1016/j.neurobiolaging.2019.10.022 31866157 PMC6995686

[34] Padilla CJ, Harrigan ME, Harris H, Profiling age-related muscle weakness and wasting: neuromuscular junction transmission as a driver of age-related physical decline. Geroscience. Published online 2021. doi:10.1007/s11357-021-00369-3 PMC8190265 33895959

[35] Owendoff G, Ray A, Bobbili P, Optimization and construct validity of approaches to preclinical grip strength testing. J Cachexia Sarcopenia Muscle. Published online 2023. 10.1002/jcsm.13300 PMC10570062 37574215

[36] Bellantuono I, de Cabo R, Ehninger D, A toolbox for the longitudinal assessment of healthspan in aging mice. Nat Protoc. 2020;15(2). doi:10.1038/s41596-019-0256-1 PMC7002283 31915391

[37] Wier CG, Crum AE, Reynolds AB, Muscle contractility dysfunction precedes loss of motor unit connectivity in SOD1(G93A) mice. Muscle Nerve. 2019;59(2). doi:10.1002/mus.26365 PMC6340745 30370671

[38] Arnold WD, Padilla Colón CJ. Maintaining Muscle Function across the Lifespan: The State of Science. Am J Phys Med Rehabil. 2020;99(12). doi:10.1097/PHM.0000000000001429 PMC7544643 32282363

[39] Ahola-Erkkilä S, Carroll CJ, Peltola-Mjösund K, Ketogenic diet slows down mitochondrial myopathy progression in mice. Hum Mol Genet. 2010;19(10). doi:10.1093/hmg/ddq076 20167576

[40] Camajani E, Feraco A, Proietti S, Very low calorie ketogenic diet combined with physical interval training for preserving muscle mass during weight loss in sarcopenic obesity: A pilot study. Front Nutr. 2022;9. doi:10.3389/fnut.2022.955024 PMC9560671 36245515

[41] Zhou Z, Kim K, Ramsey JJ, Rutkowsky JM. Ketogenic diets initiated in late mid-life improved measures of spatial memory in male mice. Geroscience. Published online 2023. doi:10.1007/s11357-023-00769-7 PMC10651563 36933143

[42] Beckett TL, Studzinski CM, Keller JN, Paul Murphy M, Niedowicz DM. A ketogenic diet improves motor performance but does not affect β-amyloid levels in a mouse model of Alzheimer’s Disease. Brain Res. 2013;1505. doi:10.1016/j.brainres.2013.01.046 PMC3825515 23415649

[43] Ari C, Murdun C, Goldhagen C, Exogenous ketone supplements improved motor performance in preclinical rodent models. Nutrients. 2020;12(8). doi:10.3390/nu12082459 PMC7468837 32824223

[44] Brownlow ML, Benner L, D’Agostino D, Gordon MN, Morgan D. Ketogenic Diet Improves Motor Performance but Not Cognition in Two Mouse Models of Alzheimer’s Pathology. PLoS One. 2013;8(9). doi:10.1371/journal.pone.0075713 PMC3771931 24069439

[45] Chen M, Bashford J, Zhou P. Motor Unit Number Estimation (MUNE) Free of Electrical Stimulation or M Wave Recording: Feasibility and Challenges. Front Aging Neurosci. 2022;14. doi:10.3389/fnagi.2022.799676 PMC8873975 35221991

[46] Gilmore KJ, Morat T, Doherty TJ, Rice CL. Motor unit number estimation and neuromuscular fidelity in 3 stages of sarcopenia. Muscle Nerve. 2017;55(5). doi:10.1002/mus.25394 27576772

[47] Campbell MJ, McComas AJ, Petito F. Physiological changes in ageing muscles. J Neurol Neurosurg Psychiatry. 1973;36(2). doi:10.1136/jnnp.36.2.174 PMC1083551 4708452

[48] Hepple RT, Rice CL. Innervation and neuromuscular control in ageing skeletal muscle. Journal of Physiology. 2016;594(8). doi:10.1113/JP270561 PMC4933121 26437581

[49] Verschueren A, Palminha C, Delmont E, Attarian S. Changes in neuromuscular function in elders: Novel techniques for assessment of motor unit loss and motor unit remodeling with aging. Rev Neurol (Paris). 2022;178(8). doi:10.1016/j.neurol.2022.03.019 35863917

[50] Zhao Z, Lange DJ, Voustianiouk A, A ketogenic diet as a potential novel therapeutic intervention in amyotrophic lateral sclerosis. BMC Neurosci. 2006;7. doi:10.1186/1471-2202-7-29 16584562 PMC1488864

[51] Streijger F, Plunet WT, Lee JHT, Ketogenic diet improves forelimb motor function after spinal cord injury in rodents. PLoS One. 2013;8(11). doi:10.1371/journal.pone.0078765 PMC3817084 24223849

[52] Thio LL, Erbayat-Altay E, Rensing N, Yamada KA. Leptin contributes to slower weight gain in juvenile rodents on a ketogenic diet. Pediatr Res. 2006;60(4). doi:10.1203/01.pdr.0000238244.54610.27 16940251

[53] Oomura Y, Hori N, Shiraishi T, Leptin facilitates learning and memory performance and enhances hippocampal CA1 long-term potentiation and CaMK II phosphorylation in rats. Peptides (NY). 2006;27(11). doi:10.1016/j.peptides.2006.07.001 16914228

[54] Irving AJ, Harvey J. Leptin regulation of hippocampal synaptic function in health and disease. Philosophical Transactions of the Royal Society B: Biological Sciences. 2014;369(1633). doi:10.1098/rstb.2013.0155 PMC3843886 24298156

[55] Ben-Shachar D, Laifenfeld D. Mitochondria, synaptic plasticity, and schizophrenia. Int Rev Neurobiol. 2004;59. doi:10.1016/S0074-7742(04)59011-6 15006492

[56] Mattson MP, Liu D. Mitochondrial potassium channels and uncoupling proteins in synaptic plasticity and neuronal cell death. Biochem Biophys Res Commun. 2003;304(3). doi:10.1016/S0006-291X(03)00627-2 12729589

[57] Li Z, Okamoto KI, Hayashi Y, Sheng M. The importance of dendritic mitochondria in the morphogenesis and plasticity of spines and synapses. Cell. 2004;119(6). doi:10.1016/j.cell.2004.11.003 15607982

[58] Baumann CW, Kwak D, Liu HM, Thompson L V. Age-induced oxidative stress: How does it influence skeletal muscle quantity and quality? J Appl Physiol. 2016;121(5). doi:10.1152/japplphysiol.00321.2016 PMC5142250 27197856

[59] Moran AL, Warren GL, Lowe DA. Soleus and EDL muscle contractility across the lifespan of female C57BL/6 mice. Exp Gerontol. 2005;40(12). doi:10.1016/j.exger.2005.09.005 16243468

[60] Ogura Y, Kakehashi C, Yoshihara T, Ketogenic diet feeding improves aerobic metabolism property in extensor digitorum longus muscle of sedentary male rats. PLoS One. 2020;15(10 October). doi:10.1371/journal.pone.0241382 PMC7598508 33125406

